# Pediatric Cutaneous Mastocytosis With Motor and Intellectual Delay

**DOI:** 10.7759/cureus.34536

**Published:** 2023-02-02

**Authors:** Yusuke Watanabe, Shinichiro Morichi, Tomoko Takamatsu, Tomonobu Ito, Gaku Yamanaka

**Affiliations:** 1 Pediatrics and Adolescent Medicine, Tokyo Medical University, Tokyo, JPN; 2 Dermatology, Tokyo Medical University, Tokyo, JPN

**Keywords:** gnb1 gene, kit gene, motor and intellectual delay, pediatrics, mastocytosis

## Abstract

Pediatric mastocytosis is a relatively rare disorder and most commonly occurs as isolated cutaneous lesions. Although autism spectrum disorders have been reported to be associated with mastocytosis, no clear association between mastocytosis and motor and intellectual delay has been reported with the exception of the case that detected de novo monoallelic mutations in the *GNB1* gene. Herein, we describe the case of a Japanese male pediatric patient aged two years and six months who had cutaneous mastocytosis accompanied by motor and intellectual delay without the presence of *GNB1* mutation.

## Introduction

Pediatric mastocytosis is a relatively rare disorder and most commonly occurs as isolated cutaneous lesions [1]. Pediatric cutaneous mastocytosis is not generally associated with motor and intellectual delay, with the exception of reported cases with* GNB1* mutation. Herein, we describe the case of a Japanese male pediatric patient aged two years and six months who had cutaneous mastocytosis accompanied by motor and intellectual delay.

This article was previously presented as a meeting abstract at the 124th Annual Meeting of the Japan Pediatric Society on April 16, 2021.

## Case presentation

**Figure 1:**
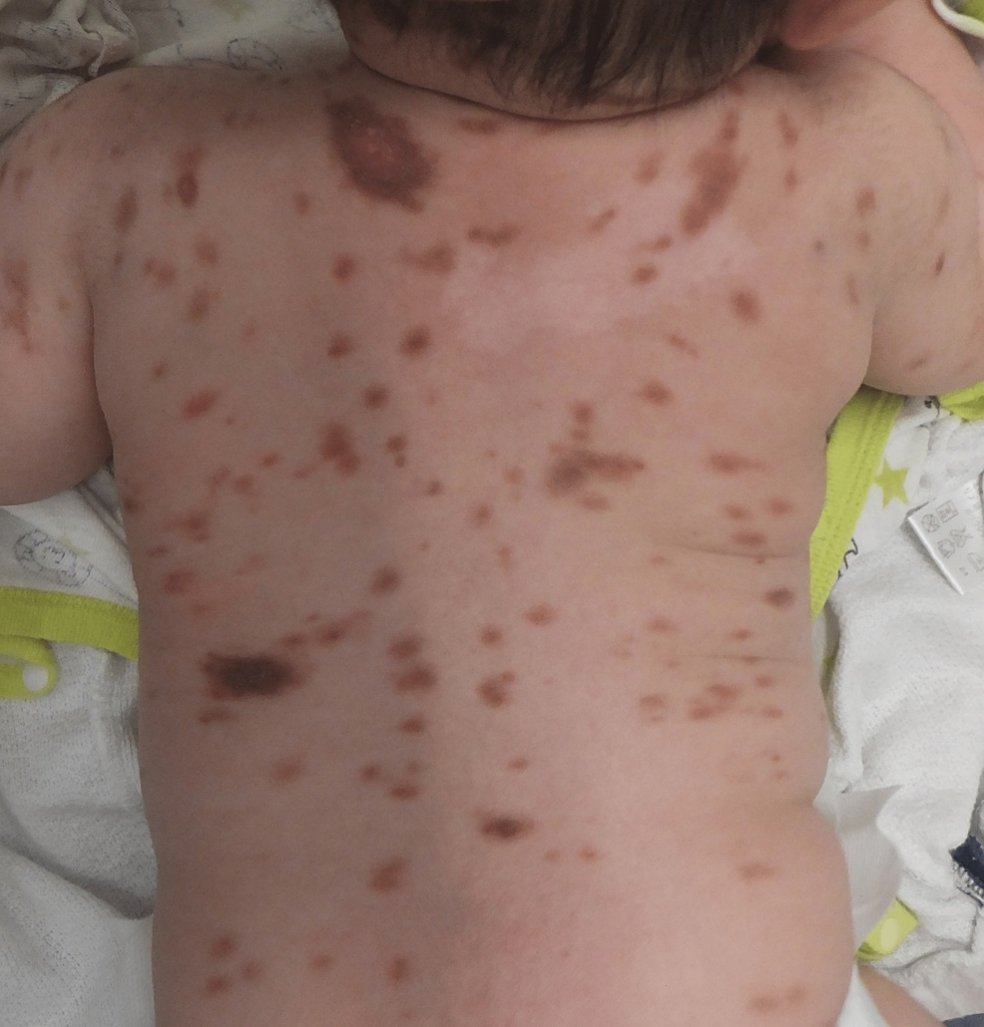
Clinical features of cutaneous mastocytosis Clinical examination revealed two nodules on the upper and left back and multiple macules scattered over the body.

The infant was born to non-consanguineous, healthy parents and was delivered vaginally at 39 weeks and three days of gestation (birth weight: 3665 g(+1.4SD), length 48.5 cm(-0.3SD), head circumference 32.3 cm(-0.7SD)). At birth, the infant did not have neonatal asphyxia or hypoxic-ischemic encephalopathy. No exposure to teratogens has been reported previously; however, a family history of ventricular septum defect in the older sister was noted. At one month of age, he was brought to the Department of Dermatology at Tokyo Medical University Hospital for a thorough examination of his skin rash. Clinical examination revealed two nodules on the upper and left back and multiple macules scattered over the body (Figure 1).

**Figure 2:**
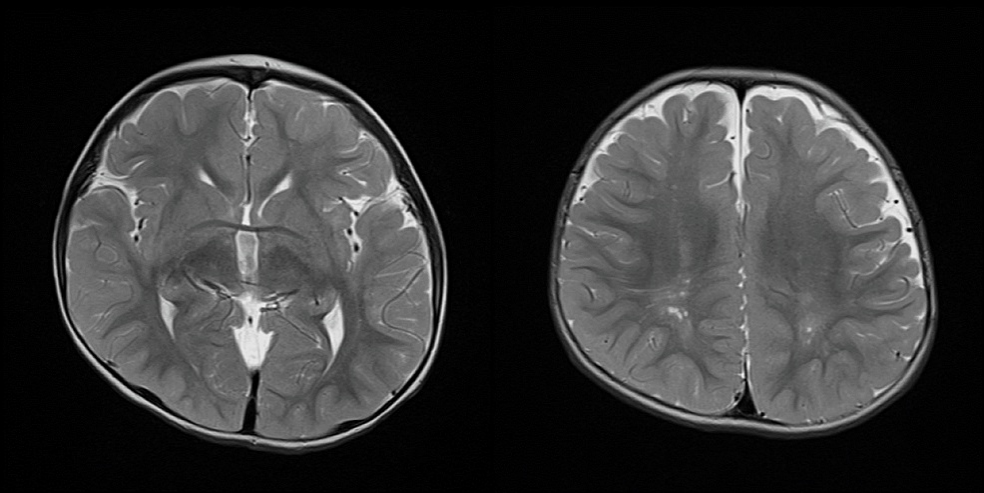
Head MRI T2WI findings at 1 year and 11 months old The image reveals high signal areas in the occipital lobe and deep frontal white matter. These signals are prominent in occipital lobe white matter and palely present in frontal lobe white matter. MRI: Magnetic resonance imaging; T2WI: T2 weighted image

Darier’s sign was positive, and on pathological examination, the number of mast cells had increased. The* KIT* gene sequenced from the skin lesion revealed a *D816V* mutation in exon 17. The patient was diagnosed with a *KIT D816V* mutation in both mastocytoma and maculopapular cutaneous mastocytosis. At nine months of age, he was observed in the Department of Pediatrics and Adolescent Medicine for motor and intellectual delay. On admission, his clinical findings included right blepharophimosis and mastocytosis. He underwent blood, urine, and spinal fluid tests and an electroencephalogram, where no obvious abnormalities were found. He also underwent chromosome analysis, which revealed a normal 46,XY karyotype. Cardiac and abdominal ultrasonography revealed only a one-mm-sized patent ductus arteriosus. Ophthalmoscopy and auditory brainstem response tests did not show any obvious abnormalities. Motor and intellectual delay was assessed using the Enjoji Scale of Infant Analytical Development, and his developmental quotient (DQ) was 56. He underwent head magnetic resonance imaging (MRI), and nonspecific findings of high signal intensity in the frontal and occipital regions on the T2 weighted image (T2WI) were apparent (Figure 2).

Sanger sequencing did not show the presence of *GNB1* mutation, which can cause *GNB1*-related neurodevelopmental disorder characterized by motor and intellectual delay, seizures, ophthalmological symptoms, and hypotonia [2]. When he was two years and four months old at the time of his last visit, he could not walk by himself or utter words. His DQ was 31, as measured by the Kyoto Scale of Psychological Development 2020, which categorized the patient as having severe motor and intellectual delay.

## Discussion

Although autism spectrum disorders, such as autistic disorder, Asperger’s disorder, or atypical autism, have been reported to be associated with mastocytosis [3], no clear association between mastocytosis and motor and intellectual delay has been reported with the exception of the case that detected de novo monoallelic mutations in the GNB1 gene, which encodes a β subunit of heterotrimeric G proteins [2]. We previously reported mastocytosis with two differential cutaneous presentations, but there was no apparent delay in motor and intellectual development at that point [4]. Subsequently, the patient started to show signs of motor and intellectual delay, but the pathogenesis was not determined in the tests that we conducted. In head MRI, nonspecific findings of high signal intensity in the frontal and occipital regions on T2WI were apparent. These findings could be related to the pathogenesis of motor and intellectual delay, e.g., myelination delay in the central nervous system, periventricular leukomalacia (PVL), or metabolic disorders such as mucopolysaccharidosis and adrenoleukodystrophy. Of these, no metabolic diseases were found to be applicable in the primary metabolic screening. Moreover, there was no suspicious history of PVL based on perinatal history. On head MRI of the patient, the frontal and occipital lobes still showed hyperintensity findings on T2WI, even though it is common for lesions in the patient over 1 and a half years old to have already changed to hypointensity due to myelination. Myelination allows rapid transmission of electrical signals at synapses and is necessary for full function in each brain region, and its delay may result in general motor and intellectual delay. In the absence of age-appropriate changes, it is possible that delayed myelination may have caused the motor and intellectual delay in this syndrome. It was recently demonstrated that mast cells selectively expressed novel MAS-related G-protein coupled receptor (GPCR) X2 [5]. *GNB1* gene encodes the G protein complex which interacts with GPCRs, and global developmental delay is observed in most patients with this mutation. Szczałuba et al. have assumed that mastocytosis in their case, and this mutation may be linked to abnormalities in GPCR downstream [2]. It has been reported that adhesion GPCRs may regulate many aspects of neural development [6]. It might be the involvement between the pathogenesis of mastocytosis in our case and motor and intellectual delay through abnormality in downstream of GPCRs. We proposed other genetic tests, including exome analysis, to determine the cause of this case’s symptom, but the parents did not give their consent and we have not yet implemented these tests. We would like to perform these tests in the future if the parents consent during the outpatient visit. In addition, it is necessary to prospectively accumulate cases with these findings and conduct genetic and other tests in order to determine the cause of cases of cutaneous mastocytosis with delayed locomotion and intelligence of unknown etiology. Regardless of the absence of *GNB1* mutations, which has been already reported for the pathology of motor and intellectual delay, the presence of motor and intellectual delay when examining pediatric mastocytosis warrants clinicians’ attention. Long-term follow-up of these patients should also be considered.

## Conclusions

In addition to GNB1 mutations, there may be other conditions that affect the association between cutaneous mastocytosis and delayed locomotion and intelligence. It is necessary to prospectively accumulate cases of mastocytosis associated with motor and intellectual delay and to actively investigate the cause of the pathology, such as conducting whole exome testing. When clinicians diagnose children with mastocytosis, they should be aware of the importance of a long-term follow-up so that they can intervene early in the event of intellectual disabilities.
